# A novel PEGylated form of granulocyte colony-stimulating factor, mecapegfilgrastim, for peripheral blood stem cell mobilization in patients with hematologic malignancies

**DOI:** 10.1186/s12885-023-11197-3

**Published:** 2023-07-24

**Authors:** Jingjing Wen, Qiaolin Zhou, Lin Shi, Fang Xu, Yiping Liu, Jing Su, Ya Zhang, Wen Qu, Jing Yue

**Affiliations:** 1 Department of Hematology, School of Medicine, Mianyang Central Hospital, University of Electronic Science and Technology of China, No 12. Changjia alley, Jingzhong Street, Fucheng district, Mianyang, 621000 China; 2 Department of Hematology of Beijing Friendship Hospital, Capital Medical University, Beijing, 100000 China

**Keywords:** Pegylated granulocyte-colony stimulating factor, Mecapegfilgrastim, Hematologic malignancies, Hematopoietic stem cell mobilization, Lymphocyte count, Predictive factors

## Abstract

**Background:**

The Pegylated recombinant human granulocyte colony stimulating factor (PEG-rhG-CSF) has longer half-life and is given once only, which is more comfortable for patients. We aimed to evaluate the efficacy of mecapegfilgrastim for hematopoietic stem cell (HSC) mobilization in patients with hematologic malignancies and to explore the potential factors related to HSC mobilization.

**Methods:**

A retrospective analysis was performed on patients who underwent HSC mobilization in the hematology department of Mianyang Central Hospital from April 2016 to November 2022. The number of CD34 + cells collected was compared between the patients receiving mecapegfilgrastim (PEG group) and those receiving recombinant human granulocyte colony-stimulating factor (rhG-CSF group), and the possible factors for mobilization failure were analyzed.

**Results:**

The success rates of collecting CD34 + cells in the PEG group and rhG-CSF group were 80.6% and 67.7%, respectively (χ = 1.444, P = 0.229). The median CD34 + cell counts were 3.62 × 10^6/kg and 2.92 × 10^6/kg (P = 0.178), respectively. After combination with plerixafor for mobilization, the median number of CD34 + cells collected in the PEG group and rhG-CSF group were 3.64 × 10^6/kg and 3.92 × 10^6/kg, respectively, with no significant difference (P = 0.754). There was no significant difference in hematopoietic cell recovery or infection between the groups (P > 0.05). Multivariate analysis showed that more than 5 cycles of chemotherapy (OR = 15.897, 95% CI: 1.766-143.127, P = 0.014), a precollection WBC count < 32 × 10^9/L (OR = 14.441, 95% CI: 2.180-95.657, P = 0.006) and a precollection to premobilization lymphocyte ratio < 1.7 (OR = 11.388, 95% CI: 2.129–60.915, P = 0.004) were independent risk factors for HSC mobilization failure.

**Conclusions:**

The HSC mobilization efficacy of mecapegfilgrastim in patients with hematologic malignancies was comparable to that of rhG-CSF, and combination with plerixafor for mobilization was feasible and effective. Patients with more than 5 cycles of chemotherapy before HSC mobilization, a precollection WBC count lower than 32 × 10^9/L, and a precollection lymphocyte count less than 1.7 times the premobilization lymphocyte count have a high probability of HSC mobilization failure.

**Supplementary Information:**

The online version contains supplementary material available at 10.1186/s12885-023-11197-3.

## Introduction

Mobilization with recombinant human granulocyte colony stimulating factor (rhG-CSF) has become the standard procedure for peripheral blood hematopoietic stem cell transplantation. Recently, the administration of rhG-CSF combined with plerixafor has become a widely used strategy to promote hematopoietic stem cell (HSC) mobilization from bone marrow to the peripheral blood for HSC transplantations [1].

The conventional form of rhG-CSF, which is nonpegylated, has a very short half-life, and multiple daily injections are required. Only one injection of pegylated G-CSF (PEG-rhG-CSF) is required, and it has a much longer half-life. PEG-rhG-CSF is a covalently bound conjugate of G-CSF and polyethylene glycol molecules, which results in its prolonged duration of action [2]. As a single dose of PEG-rhG-CSF is sufficient for HSC mobilization and is associated with less pain [3], it has become a more convenient option for outpatients. Some studies have demonstrated the convenience, efficiency, and safety of mobilizing HSCs with PEG-rhG-CSF at a fixed dose of 6 mg or 12 mg [4–7]. The study by Bruns et al. [8] concluded that mobilization with 12 mg PEG-rhG-CSF significantly shortened the mobilization duration compared with the 6 mg dose. A meta-analysis published by Kuan JW et al. [4] showed that CD34 + cell counts in the PEG-rhG-CSF 12 mg group reached higher peak levels. Xiao Ding et al. [9] also found that mobilization with 12 mg PEG-rhG-CSF further shortened the time to recovery of leukocytes and platelets. Furthermore, the total cost of mobilization and apheresis using PEG-rhG-CSF or conventional rhG-CSF was comparable [9].

The NCCN Clinical Practice Guidelines (Version 1.2022) [10] recommend HSC mobilization with pegfilgrastim for autologous donors, and an FDA-approved biosimilar is an appropriate substitute for pegfilgrastim. The commercially pegylated rhG-CSF is pegfilgrastim. Mecapegfilgrastim is a biosimilar of pegfilgrastim and has been developed in China. However, there are limited data on mecapegfilgrastim use for HSC mobilization. Therefore, we performed a retrospective study to evaluate HSC mobilization outcomes with mecapegfilgrastim versus conventional rhG-CSF for patients with hematologic malignancies. Factors related to HSC mobilization failure were also explored.

## Patients and methods

### Patients

A retrospective analysis of all patients mobilized and collected from peripheral HSCs using rhG-CSF or mecapegfilgrastim in the Department of Hematology of the Mianyang Central Hospital between April 2016 and November 2022 was performed. In this study, we included acute leukemia, lymphoma and plasma cell disease patients. Inclusion criteria were as follows: (1) age 14–70 years and (2) definite indications for autologous hematopoietic stem cell transplantation (auto-HSCT). Exclusion criteria: (1) age < 14 years old or > 70 years; (2) severe cardiopulmonary insufficiency; and (3) no indication for auto-HSCT.

### Mobilization and collection of stem cells

Mobilization with mecapegfilgrastim or rhG-CSF was performed as decided by the patients. However, mecapegfilgrastim is not approved for patients with myeloid malignancies based on drug package instructions. PEG group: Mecapegfilgrastim (Jiangsu Hengrui Pharmaceutical Co., Ltd.) was given as a single dose of 12 mg. rhG-CSF group: rhG-CSF (Rebai, Qilu Pharmaceutical Co., Ltd.) was given twice at a dose of 10 µg/kg/day for 4 to 7 days according to the efficacy of collection.

Mobilization may be combined with chemotherapy and/or plerixafor (Mozobil, Sanofi (Beijing) Pharmaceutical Co., Ltd.). Plerixafor was administered 11 h prior to harvest, with a fixed dose of 20 mg for patients weighing ≤ 83 kg and 0.24 mg/kg for those weighing > 83 kg. HSCs were harvested on Day 4 after G-CSF administration. The harvesting period was 1 to 3 days according to the collection results.

### Evaluation and definitions

According to the Diagnostic and Therapeutic Criteria of Hematological Diseases (version 4) [11], the treatment response of acute leukemia and lymphoma before mobilization was assessed as complete response (CR) or partial response (PR), and the treatment response of plasma cell disease was assessed as CR, very good partial response (VGPR) or PR. The mobilization efficacy was evaluated as failure, standard and optimal collection according to the total number of collected CD34 + cells per kilogram (kg) of body weight [12]. Failure of mobilization was defined as the collection of < 2 × 10^6 CD34 + cells/kg. Standard mobilization was defined as the collection of 2–5 CD34 + cells/kg. Optimal mobilization was defined as the collection of ≥ 5 × 10^6 CD34 + cells/kg. The success rate of mobilization was defined as the sum of the standard and optimal collection rates. HSC collection by apheresis was started and continued daily for up to 3 days or until HSC collection was successful. Apheresis was performed using the FRESENIUS KABI COM.TEC.

The days of hematopoietic cell implantation, infections, amount of blood transfusion and duration of hospitalization for auto-HSCT were followed up. The time of neutrophil implantation was defined as the time to reach an absolute neutrophil count of 0.5 × 10^9/L on 3 consecutive days after autologous hematopoietic cell transfusion. The time of platelet implantation was defined as the time to reach a platelet count of 20 × 10^9/L for at least 7 unsupported consecutive days and at least 48 h after the last platelet transfusion.

### Statistical analysis

All statistical analyses were performed with SPSS version 26.0. Measurement data conforming to a normal distribution are represented by the mean ± standard deviation. Nonnormal measurement data are expressed as the median (range). Categorical variables were analyzed by the chi-square or Fisher exact test, and continuous variables were analyzed by the t test or nonparametric test if the data were not normally distributed. Multivariate analysis was carried out by a logistic regression model. A two-tailed P < 0.05 was regarded as significant.

## Results

### Clinical characteristics

A total of 67 patients were included in this study, including 40 males and 27 females, with a median age of 50 years (15–66 years). There were 12 cases of acute leukemia (9 cases of myeloid system, 3 cases of lymphoid system), 21 cases of lymphoma (17 cases of non-Hodgkin’s lymphoma (NHL), 4 cases of Hodgkin’s lymphoma), 34 cases of plasma cell disease (33 cases of multiple myeloma (MM), and 1 case of systemic amyloidosis light chain).

Thirty-six patients were included in the PEG group, and these patients received mecapegfilgrastim alone (n = 12), mecapegfilgrastim and plerixafor (n = 22), mecapegfilgrastim and chemotherapy (n = 1), and mecapegfilgrastim plus chemotherapy and plerixafor (n = 1). There were thirty-one patients in the rhG-CSF group, and these patients received rhG-CSF alone (n = 8), rhG-CSF and plerixafor (n = 9), rhG-CSF and chemotherapy (n = 10), and rhG-CSF plus chemotherapy and plerixafor (n = 4). There was no significant difference between the PEG group and the rhG-CSF group in clinical baseline characteristics, including age, sex, disease classification, premobilization disease status, and combination with plerixafor (P > 0.05), as shown in Table 1.

**Table 1:** Comparison of baseline clinical characteristics between the PEG and rhG-CSF groups

Clinical characteristic	PEG group N = 36 cases	rhG-CSF group N = 31 cases	Statistical value	P value
Age (year)	52(25–66)	47(15–63)	0.944	0.345
Sex			0.567	0.451
Male	63.9% (23/36)	54.8% (17/31)		
Female	36.1% (13/36)	45.2% (14/31)		
Classification of diseases			9.295	0.010
Acute Leukemia	5.6% (2/36)	32.3% (10/31)		
Lymphoma	30.6% (11/36)	32.3% (10/31)		
Plasma cell disease	63.9% (23/36)	35.5% (11/31)		
ECOG score			0.105	0.746
0–1	70.6% (24/34)	74.2% (23/31)		
2–4	29.4% (10/34)	25.8% (8/31)		
BMI (kg/m^2^)	23.72 ± 3.18	24.98 ± 4.48	1.339	0.185
Lymphoma risk stratification			0.276	1.000
Low-intermediate	36.4% (4/11)	40.0% (4/10)		
High	27.3% (3/11)	30.0% (3/10)		
Unknown	36.4% (4/11)	30.0% (3/10)		
MM ISS stage			1.168	0.565
I	36.4% (8/22)	36.4% (4/11)		
II	50.0% (11/22)	36.4% (4/11)		
III	13.6% (3/22)	27.3% (3/11)		
Number of chemotherapy cycles			3.656	0.056
< 5	55.6% (20/36)	32.3% (10/31)		
≥ 5	44.4% (16/36)	67.7% (21/31)		
Lenalidomide exposure			6.804	0.009
Yes	50.0% (18/36)	19.4% (6/31)		
No	50.0% (18/36)	80.6% (25/31)		
Premobilization disease status			0.083	0.773
Newly diagnosed	77.8% (28/36)	80.6% (25/31)		
Remission after recurrence	22.2% (8/36)	19.4% (6/31)		
Premobilization disease response			0.844	0.656
CR	50.0% (15/30)	62.5% (15/24)		
VGPR	33.3% (10/30)	25.0% (6/24)		
PR	16.7% (5/30)	12.5% (3/24)		
Combination chemotherapy			14.374	0.000
Yes	5.6% (2/36)	45.2% (14/31)		
No	94.4% (34/36)	54.8% (17/31)		
Combination plerixafor			3.229	0.072
Yes	63.9% (23/36)	41.9% (13/31)		
No	36.1% (13/36)	58.1% (18/31)		

rhG-CSF, recombinant human granulocyte colony stimulating factor; PEG group, Mobilization of hematopoietic stem cells with mecapegfilgrastim; BMI, body mass index; MM, multiple myeloma; CR, complete response, PR, partial response, VGPR, very good partial response

The proportion of patients with lenalidomide exposure before mobilization was higher in the PEG group than in the rhG-CSF group (P = 0.009). A total of 87.5% of patients with plasma cell disease in the PEG group were exposed to lenalidomide, which was higher than that in the rhG-CSF group (P = 0.010). The combination chemotherapy rate in the rhG-CSF group was significantly higher than that in the PEG group (P = 0.0001). In the rhG-CSF group, 14 patients were mobilized by combination chemotherapy, including 4 patients with acute leukemia, 7 patients with lymphoma and 3 patients with plasma cell disease. There were 2 patients in the PEG group, including 1 patient with lymphoma and 1 patient with plasma cell disease. The difference in the proportions of patients receiving combination chemotherapy may be related to the different disease distributions between the groups. Regarding the wide use of plerixafor, it should be noted that patients gradually preferred to choose static mobilization strategies combining plerixafor rather than chemotherapy. Whether in the overall study population, the PEG group or rhG-CSF group, combination chemotherapy had no significant impact on the success of HSC collection (P > 0.05, Supplementary Table 1).

### Efficacy-Mecapegfilgrastim vs. rhG-CSF

The success rate of the PEG group was 80.6%, slightly higher than the 67.7% success rate of the rhG-CSF group, but the difference was not significant (χ = 1.444, P = 0.229).

The median number of mononuclear cells (MNCs) collected was 16.83 × 10^8/kg in the PEG group and 16.00 × 10^8/kg in the rhG-CSF group (P = 0.505), and the median CD34 + cell counts were 3.62 × 10^6/kg and 2.92 × 10^6/kg, respectively (P = 0.178). For the patients mobilized with plerixafor, the success rate of mobilization was 82.6% (19/23) in the PEG group and 84.6% (11/13) in the rhG-CSF group (P = 1.000). The median number of CD34 + cells collected did not differ significantly between the PEG group and the rhG-CSF group (3.64 × 10^6/kg vs. 3.92 × 10^6/kg, P = 0.754). The median precollection WBC counts were 52.31 × 10^9/kg in the group treated with mecapegfilgrastim and 40.70 × 10^9/kg in the group treated with rhG-CSF, and the difference was statistically significant (P = 0.007). There was no significant difference in the lymphocyte/monocyte count ratio, hemoglobin level or platelet level between the PEG group and the rhG CSF group before mobilization and collection (P > 0.05).

Forty-one patients completed the auto-HSCT process. There was no significant difference in hematopoietic recovery, infection, blood transfusion volume or hospitalization duration between the PEG group and the rhG-CSF group (P > 0.05), as shown in Table 2.

**Table 2:** Comparison of mobilization efficacy in the PEG group and the rhG-CSF group

Characteristic	PEG group N = 36 cases	rhG-CSF group N = 31 cases	Statistical value	P value
Premobilization WBC (×10^9/L)	4.67(1.19–9.89)	4.67(1.68–10.93)	0.692	0.489
Premobilization lymphocyte/monocyte count ratio	1.91(0.84–5.29)	2.25(0.15–6.19)	0.953	0.341
Premobilization HGB (g/L)	122(59–159)	122(61–139)	0.704	0.481
Premobilization PLT (×10^9/L)	176(74–306)	169(7-498)	0.535	0.593
Precollection WBC (×10^9/L)	52.31 ± 18.26	40.70 ± 15.30	2.794	0.007
Precollection lymphocyte/monocyte count ratio	0.55(0.28–8.14)	0.59(0.19–4.22)	0.747	0.455
Precollection HGB (g/L)	114 ± 19	112 ± 20	0.397	0.693
Precollection PLT (×10^9/L)	129(49–260)	126(68–459)	0.157	0.875
Collection of MNC counts (10^8/kg)	16.83(6.88–47.60)	16.00(3.48–36.61)	0.666	0.505
Collection of CD34 + cell counts (10^6/kg)	3.62(0.32–13.26)	2.92(0.05–9.85)	1.346	0.178
Mobilization efficacy			1.451	0.484
Failure	19.4% (7/36)	32.3% (10/31)		
Standard	52.8% (19/36)	45.2% (14/31)		
Optimal	27.8% (10/36)	22.6% (7/31)		
Collection of CD34 + cell counts with plerixafor (10^6/kg)	3.64(0.32–13.26)	3.92(0.40–9.17)	0.313	0.754
Time of neutrophil implantation (days)	9.96 ± 0.87	9.64 ± 1.22	0.959	0.344
Time of platelet implantation (days)	11.88 ± 2.41	11.79 ± 1.76	0.135	0.893
Occurrence of infection			0.000	1.000
Yes	77.8% (21/27)	78.6% (11/14)		
No	22.2% (6/27)	21.4% (3/14)		
Transfusion of red blood cell (U)	0(0–6)	0(0–3)	0.159	0.873
Transfusion of PLT (therapeutic volumes)	2(0–5)	1.5(1–4)	0.044	0.965
Hospitalization duration (days)	23(19–30)	24(19–39)	1.112	0.266

rhG-CSF, recombinant human granulocyte colony stimulating factor; PEG group, Mobilization of hematopoietic stem cells with mecapegfilgrastim; WBC, white blood cell; HGB, hemoglobin; PLT, platelet

### Mecapegfilgrastim in MM

Among the thirty-three cases of MM, 22 patients were treated with mecapegfilgrastim mobilization and 11 underwent rhG-CSF mobilization. There was no significant difference between the PEG group and rhG-CSF group in terms of baseline clinical characteristics, including sex, age, BMI, ISS stage, ECOG score, exposure to lenalidomide, mobilization strategies (with/without chemotherapy), combination with plerixafor, premobilization disease status, and number of chemotherapy cycles before mobilization (P > 0.05).

A total of 90.9% of the patients with MM underwent standard collection, of whom 36.4% achieved optimal collection. The rates of successful mobilization in the PEG group and rhG CSF group were 90.9% and 90.9%, respectively, and the optimal HSC collection rates were 31.8% and 45.5%, respectively. There was no significant difference in the rate of mobilization between the groups, with P values of 1.000 and 0.471, respectively. The median CD34 + cell count was 3.87 (0.65–13.26) ×10^6/kg in the PEG group and 4.33 (1.27–9.85) ×10^6/kg in the rhG-CSF group, with no significant difference (Z = 0.420, P = 0.674). The median number of MNCs collected in the PEG group was 17.26 (6.88–42.84) × 10^8/kg in the PEG group and 16.77 (5.47–30.05) × 10^8/kg in the rhG-CSF group, with no significant difference (Z = 0.306, P = 0.760).

Twenty-one patients with MM underwent auto-HSCT at our center, including 16 in the PEG group and 5 in the rhG-CSF group. The median time to platelet implantation was 12.1 ± 2.0 days in the PEG group compared with 11.4 ± 1.3 days in the rhG-CSF group (t = 0.759, P = 0.457). The median time to neutrophil implantation was 10 (9~12) days in the PEG group compared with 10 (10~12) days in the rhG-CSF group (Z = 0.445, P = 0.656). The duration of hospitalization was 21.8 ± 2.4 days in the PEG group and 23.0 ± 3.5 days in the rhG-CSF group (t = 0.865, P = 0.398). There was no significant difference between the PEG group and the rhG-CSF group in terms of infection, red blood cell transfusion or platelet transfusion, with P values of 1.000, 0.307 and 0.101, respectively.

### Risk factors for HSC mobilization

The success rates of HSC mobilization were 91.2%, 61.9%, and 50.0% for plasma cell disease, lymphoma, and acute leukemia, respectively (χ = 10.812, P = 0.004).

Compared with those who for whom stem cell collection failed, those for whom stem cell collection succeeded had fewer chemotherapy cycles, higher precollection WBC counts, higher precollection absolute lymphocyte counts, higher precollection absolute monocyte counts, higher precollection PLT counts, a higher precollection to premobilization monocyte ratio, and a higher precollection to premobilization lymphocyte ratio, with statistically significant differences (P < 0.05). However, there were no significant differences in HSC mobilization according to age, sex, BMI, disease stage, disease risk stratification, ECOG score, lenalidomide exposure, combination chemotherapy, long-acting G-CSF mobilization, premobilization HGB, or precollection HGB (P > 0.05).

ROC curves were used to determine the optimal cutoff for each of these parameters. Six variables were included in the multivariate analysis, including disease classification (plasma cell disease and nonplasma cell disease), number of chemotherapy cycles ≥ 5, a precollection WBC count < 32 × 10^9/L, a precollection to premobilization monocyte ratio < 9.0, a precollection to premobilization lymphocyte ratio < 1.7, and a precollection PLT count < 100 × 10^9/L. Multivariate analysis showed that more than 5 chemotherapy cycles (OR = 15.897, 95% CI: 1.766-143.127, P = 0.014), a precollection WBC count < 32 × 10^9/L (OR = 14.441, 95% CI: 2.180-95.657, P = 0.006) and a precollection to premobilization lymphocyte ratio < 1.7 (OR = 11.388, 95% CI: 2.129–60.915, P = 0.004) were independent risk factors for HSC mobilization failure.

## Discussion

### Mecapegfilgrastim and rhG-CSF

We retrospectively analyzed the efficacy of mecapegfilgrastim and recombinant human granulocyte colony-stimulating factor for hematopoietic stem cell mobilization in patients with hematologic malignancies.

Lavinia Lipan et al. [7] used PEG-rhG-CSF to collect HSCs in lymphoma patients with a success rate of 90.9%. Wang Ting et al. [13] used PEG-rhG-CSF combined with chemotherapy to collect HSCs in MM and lymphoma patients with a success rate of 92.1%. The success rates were both higher than the success rate of 80.6% in the PEG-rhG-CSF group in our study. The reason may be related to the different disease entities in that patients with acute leukemia were included in our study. For patients with plasma cell disease only, the success rate of HSC mobilization was 90.9%, similar to the above reports. The different mobilization strategies used may be responsible for the difference.

Ding X et al. [9] reported that PEG-rhG-CSF is superior to rhG-CSF for HSC mobilization. Some studies have also [14, 15] suggested that short-acting G-CSF (filgrastim) is superior to long-acting G-CSF (pegfilgrastim) in HSC mobilization. However, more studies have reported similar efficacy of hemopoietic stem cell mobilization with PEG-rhG-CSF compared with rhG-CSF. Shao Shan et al. [16] reported no significant differences in the quantity of CD34 + cells, MNC counts or time to hematopoietic recovery between PEG-rhG-CSF and rhG-CSF mobilization protocols in patients with relapsed refractory malignant lymphomas. Moreover, the application of PEG-rhG-CSF significantly reduced patient costs. Lipan L et al. [7] used long-acting G-CSF (pegfilgrastim) and short-acting G-CSF (filgrastim) in combination with chemotherapy to mobilize HSCs in 32 patients with lymphoma. They found similar mobilization efficacy in the two groups. A meta-analysis [4] included five clinical trials comparing the efficacy of PEG-rhG-CSF versus conventional rhG-CSF for HSC mobilization. Data from two studies showed no significant difference in the mobilization success rate between the groups. Data from the other three studies showed that there was no significant difference in the quantity of CD34 + cells, the incidence of adverse events, or the days of neutrophil and platelet implantation between the groups [4]. In our study, no significant differences were found in the mobilization success rate, MNC counts or quantity of CD34 cells between the mecapegfilgrastim group and the rhG-CSF group. This result suggested that the mobilization efficacy in patients with hematologic malignancies was similar to that of conventional rhG-CSF. In addition, there were no significant differences between the groups in the time to hematopoietic recovery, incidence of infection, or hospitalization duration during auto-HSCT in our study. This finding also suggests that there is no difference in the quality of HSCs obtained with mecapegfilgrastim and conventional rhG-CSF.

There are few studies on the mobilization of long-acting G-CSF combined with plerixafor. Partanen A et al. [17] reported that HSC collection was safe and effective in 92% of NHL patients after mobilization with pegfilgrastim combined with plerixafor and chemotherapy. A study by Watts NL et al. [5] showed that the success rate of pegfilgrastim combined with plerixafor was as high as 95%. Among the patients mobilized with plerixafor in this study, the mobilization success rate was 82.6% in the mecapegfilgrastim group, which was not significantly different from that in the rhG-CSF group. This suggests that the mobilization of mecapegfilgrastim plus plerixafor is feasible and effective.

### Mecapegfilgrastim in MM

In this study, patients undergoing HSC mobilization treated with mecapegfilgrastim were predominantly patients with MM. Lenalidomide exposure was more common in the mecapegfilgrastim group than in the rhG-CSF group. Therefore, we analyzed the HSC mobilization efficacy of mecapegfilgrastim in MM patients.

In this study, HSCs were successfully mobilized by mecapegfilgrastim or conventional rhG-CSF in more than 90% of patients with MM. Wang Ting et al. [13] reported an HSC mobilization success rate of 90.6% in a plasma cell disease group using PEG-rhG-CSF. Ivetta Danylesko et al. [6] reported a novel longacting G-CSF (lipegfilgrastim) for HSC mobilization in MM, and the mobilization success rate was 87.5%. The results reported above are similar to those of our study. In MM patients, we found no difference in HSC mobilization efficacy between mecapegfilgrastim and rhG-CSF. Muhammad Bilal Abid et al. [18] also demonstrated no significant difference in the median number of HSCs collected or time to neutrophil implantation between a pegfilgrastim group and a filgrastim group in MM patients who had received a novel agent-based induction chemotherapy. Skopec B et al. [19] found that the median number of collected HSCs was 5.05 × 10^6/kg in the filgrastim group and 4.66 × 10^6/kg in the pegfilgrastim group (P = 0.428). The median times to neutrophil and platelet implantation were similar in the groups.

It has also been reported that the mobilization of PEG-rhG-CSF is superior to that of rhG-CSF. A real-world study from China evaluated the efficacy and cost of pegfilgrastim for mobilization. PEG-rhG-CSF (pegfilgrastim) mobilization yielded a significantly higher median number of collected CD34 + cells than filgrastim mobilization (5.56 vs. 4.82 × 10^6/kg, P = 0.038). This result suggested that pegfilgrastim improved the outcomes of mobilization. The number of CD34 + cells reported was higher than that in our study. This may be related to the different mobilization protocols combining pegfilgrastim or filgrastim with high-dose chemotherapy. Only a few patients in our study were mobilized by combination chemotherapy.

### Predictors of poor HSC mobilization

The failure of HSC mobilization is an important problem in HSCT, and the identification of patients at high risk of failing mobilization may help to implement mobilization stratification strategies.

In our study, univariate analysis showed that patients with lymphoma, a lower precollection absolute monocyte count and a lower precollection PLT count (< 100 × 10^9/L) had a higher rate of HSC mobilization failure, as reported in the literature [20–23]. However, these results were not verified by multivariate analysis.

Olivieri J et al. [22] found that an increasing number of full chemotherapy courses was an independent predictive factor for mobilization failure. Bhamidipati P et al. [24] retrospectively analyzed 706 eligible patients with hematologic malignancies. The study found that the median number of chemotherapy cycles was 5 for successful mobilization, and the median number of chemotherapy cycles was 8.5 for failed mobilization. Zhang Hong et al. [25] showed that autologous HSC mobilization was more successful when the number of premobilization chemotherapy cycles was less than seven. Multivariate analysis in our study identified that ≥ 5 chemotherapy cycles was a risk factor for HSC mobilization failure. This finding suggested that it is better for patients to complete autologous HSC mobilization within 5 cycles of chemotherapy to achieve successful HSC collection.

Olivieri J et al. [22] reported that a low premobilization WBC count (< 5 × 10^9/L) was an independent predictive factor for mobilization failure. A report from a South African center also showed that a low white cell count (WCC) at collection (WCC < 9 × 10^9/L) was one of the risk factors for poor mobilization [26]. BAO Wen et al. [27] showed that there was a positive correlation of the WBC count in peripheral blood before apheresis with the collection of CD34 + cells, and a WBC count > 10.68 × 10^9/L before apheresis usually predicted successful mobilization. In our study, multivariate analysis showed that a precollection WBC count < 32 × 10^9/L was an independent risk factor for HSC mobilization failure, similar to previously reported results. This finding demonstrated that the precollection WBC counts in peripheral blood were closely related to the collection of CD34 + cells, and HSC mobilization was more likely to fail when the precollection WBC count was lower than 32 × 10^9/L.

In apheresis-based mononuclear cell (MNC) collections, MNCs include lymphocytes, monocytes, and pluripotent CD34 + HSCs. A study [28] found that a higher absolute lymphocyte count and higher relative monocyte count premobilization showed a positive correlation with the CD34 + cell counts on day + 5. In our study, multivariate analysis showed that a precollection to premobilization lymphocyte ratio less than 1.7 was an independent risk factor for HSC mobilization failure. This result suggested that apheresis failure was more likely if the absolute precollection lymphocyte count did not rise above 1.7 after mobilization.

The PB CD34 + cell count measured by flow cytometry was the most common parameter used to initiate hematopoietic progenitor cell apheresis collection. As more rapidly available biomarkers, leucocyte and mononuclear cell counts are recommended to evaluate the timing of HSC apheresis and to predict HSC mobilization [29]. The prediction of successful HSC mobilization based on the precollection WBC count and the precollection to premobilization lymphocyte ratio may be a feasible approach in the absence of timely monitoring of the CD34 cell count.

## Conclusions

In conclusion, we used mecapegfilgrastim to mobilize HSCs in patients with hematologic malignancies. The efficacy of mecapegfilgrastim for HSC mobilization was proven to be comparable to that of conventional rhG-CSF. We found that more than 5 cycles of chemotherapy before HSC mobilization, a precollection WBC count lower than 32 × 10^9/L or a precollection to premobilization lymphocyte ratio lower than 1.7 were related to poor mobilization. Finally, some limitations should be noted. This was a retrospective study with a relatively small number of patients and a wide range of 95% CIs for risk factors. Thus, the conclusions need to be further confirmed by expanding the number of patients or by performing prospective cohort studies. In addition, mecapegfilgrastim has not been used for mobilization in patients with acute myeloid leukemia, primarily based on the indications not approved for myeloid malignancies in the package insert. Therefore, the classification of diseases in the PEG group and rhG-CSF group was not exactly the same. Future randomized controlled studies are needed to evaluate the efficacy of mecapegfilgrastim mobilization in patients with hematologic malignancies.

## Electronic supplementary material

Below is the link to the electronic supplementary material.

Supplementary Material 1

## Data Availability

The data that support the findings of this study are available from the corresponding author upon reasonable request.

## Abbreviations

rhG-CSF: recombinant human granulocyte colony stimulating factor
G-CSF: granulocyte colony stimulating factor
PEG-rhG-CSF: Pegylated recombinant human granulocyte colony stimulating factor
HSC: hematopoietic stem cell
WBC: white blood cell
HGB: hemoglobin
PLT: platelet
BMI: body mass index
MM: multiple myeloma
CR: complete response
PR: partial response
VGPR: very good partial response
auto-HSCT: autologous hematopoietic stem cell transplantation
MNC: mononuclear cell
