# Supplementary material for: A novel PEGylated form of granulocyte colony-stimulating factor, mecapegfilgrastim, for peripheral blood stem cell mobilization in patients with hematologic malignancies
Source: BMC Cancer. 2023 Jul 24;23:694. doi: 10.1186/s12885-023-11197-3 (PMC10364371; doi:10.1186/s12885-023-11197-3)
Supplement: Supplementary file 1 — Supplementary Material 1 [file 12885_2023_11197_MOESM1_ESM.docx]

Supplementary Table 1 Impact of combination chemotherapy on HSC collection

| Characteristic | Mobilization efficacy in all patients | | Mobilization efficacy in PEG group | | Mobilization efficacy in rhG-CSF group | |
| --- | --- | --- | --- | --- | --- | --- |
|  | Success | Failure | Success | Failure | Success | Failure |
| Combination chemotherapy |  |  |  |  |  |  |
| Yes | 68.8%（11/16） | 31.3%（5/16） | 100%（2/2） | 0%（0/2） | 64.3%（9/14） | 35.7%（5/14） |
| No | 76.5%（39/51） | 23.5%（12/51） | 79.4%（27/34） | 20.6%（7/34） | 70.6%（12/17） | 29.4%（5/17） |
| χ value | 0.084 | | - | | 0 | |
| P value | 0.772 | | 1.000 | | 1.000 | |

rhG-CSF, recombinant human granulocyte colony stimulating factor; PEG group, Mobilization of hematopoietic stem cells with mecapegfilgrastim.
